# Supplementary material for: PEEK–WC-Based Mixed Matrix Membranes Containing Polyimine Cages for Gas Separation
Source: Molecules. 2021 Sep 13;26(18):5557. doi: 10.3390/molecules26185557 (PMC8470936; doi:10.3390/molecules26185557)
Supplement: Supplementary file 1 [file molecules-26-05557-s001.zip › molecules-1351825-supplementary.pdf]

molecules

SUPPLEMENTARY INFORMATION

PEEK-WC-based Mixed Matrix Membranes Containing Polyimine Cages for Gas Separation

Marcello Monteleone [1], Riccardo Mobili [2], Chiara Milanese [2], Elisa Esposito [1], Alessio Fuoco [1,*], Sonia La Cognata[2,*], Valeria Amendola [2] and Johannes C. Jansen [1]

[1] Institute on Membrane Technology, National Research Council of Italy (CNR-ITM), via P. Bucci 17/C, Rende (CS), 87036, Italy: m.monteleone@itm.cnr.it (M.M) e.esposito@itm.cnr.it (E.E), jc.jansen@itm.cnr.it (J.C.J.)

[2] Dipartimento di Chimica, Università di Pavia, via Taramelli 12, I-27100 Pavia, Italy: riccardo.mobili01@universitadipavia.it (R.M.), chiara.milanese@unipv.it (C.M.), valeria.amendola@unipv.it (V.A.)

* Correspondence: a.fuoco@itm.cnr.it (A.F.), sonia.lacognata01@universitadipavia.it (S.L.C.)

Index

1. Characterization of the Cages 2

1.1 ^{1}H-NMR spectra 2

1.2 FTIR-ATR spectra 4

2. Differential scanning calorimetric (DSC) measurements 6

1. Characterization of the Cages

1.1 ¹H-NMR spectra

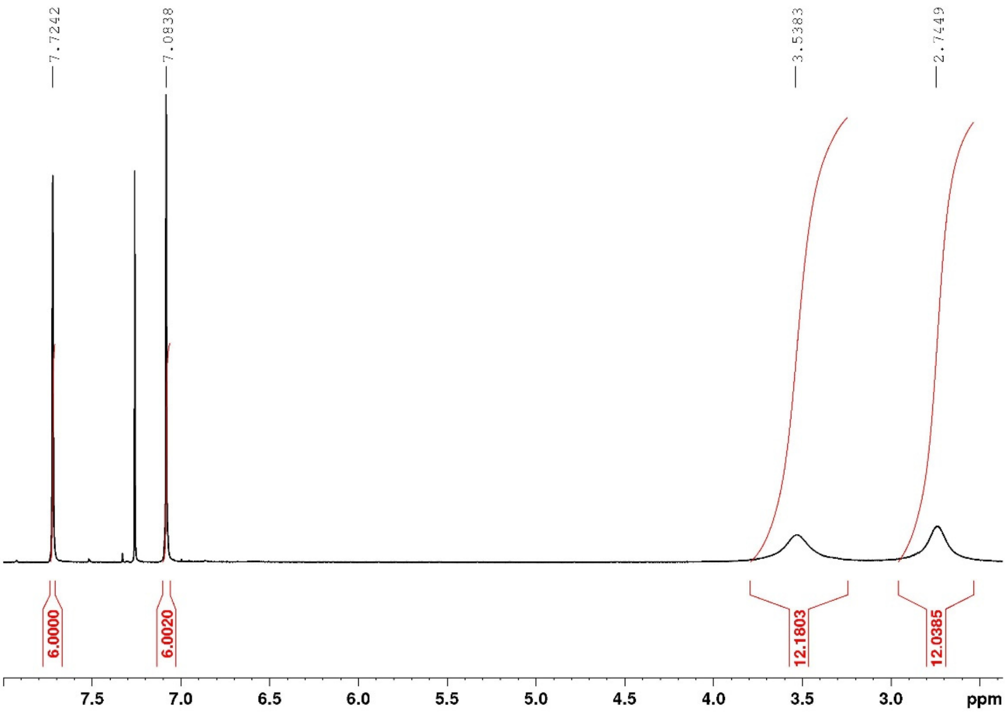

Figure S1. ¹H–NMR spectrum of Fura in $CDCl_3$.

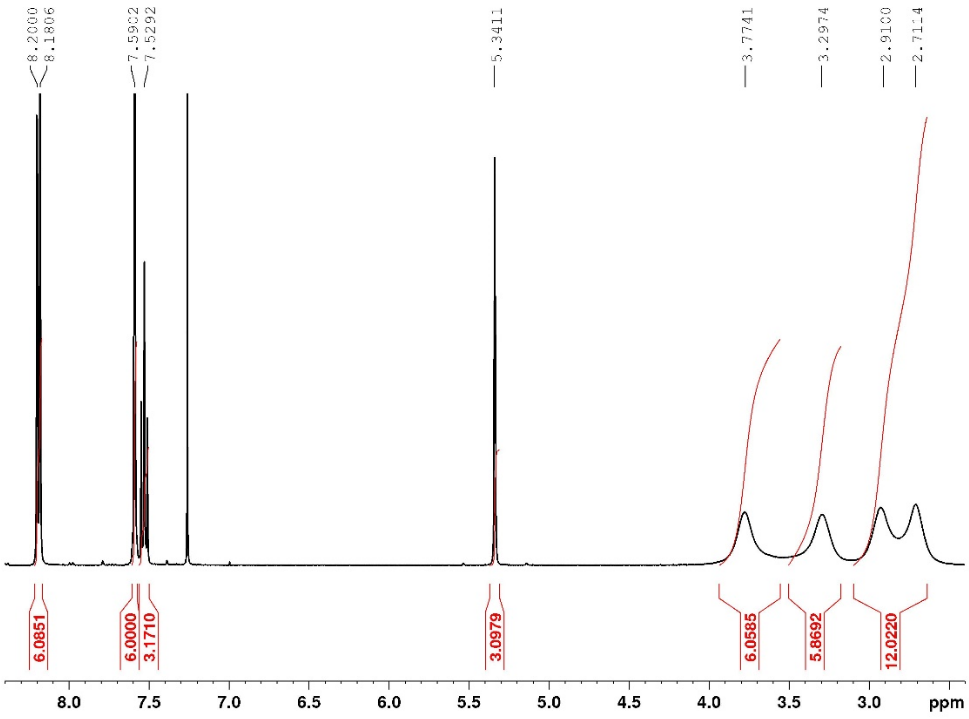

Figure S2. ¹H–NMR spectrum of m-xy in $CDCl_3$..

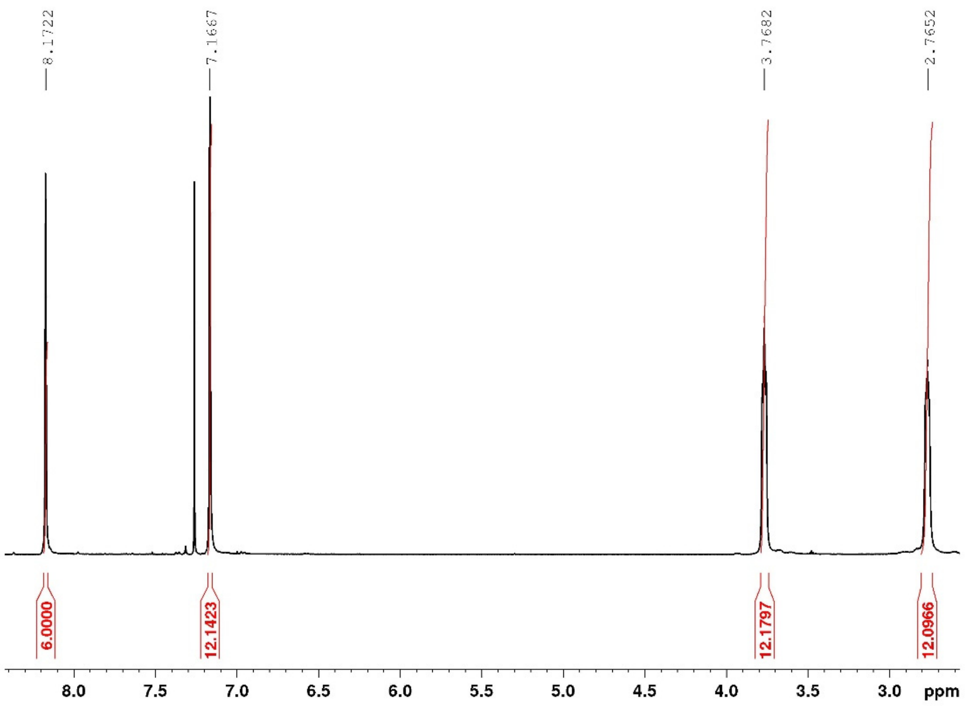

Figure S3. ^{1}H–NMR spectrum of p-xy in $CDCl_3$..

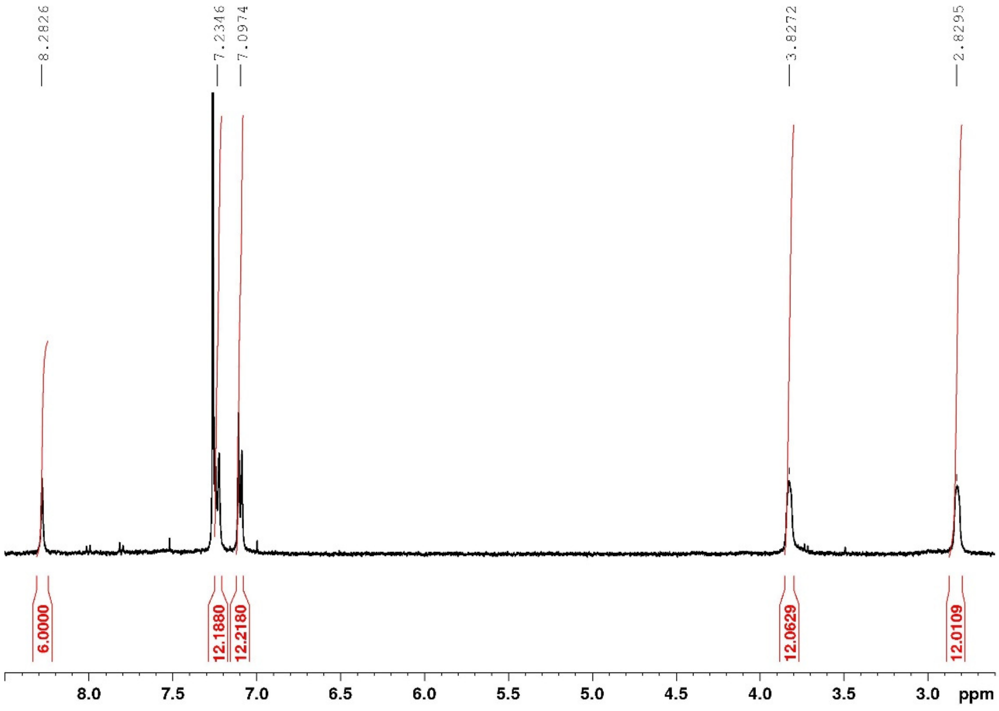

Figure S4. ^{1}H–NMR spectrum of the diphenyl cage in $CDCl_3$.

1.2 FTIR-ATR spectra

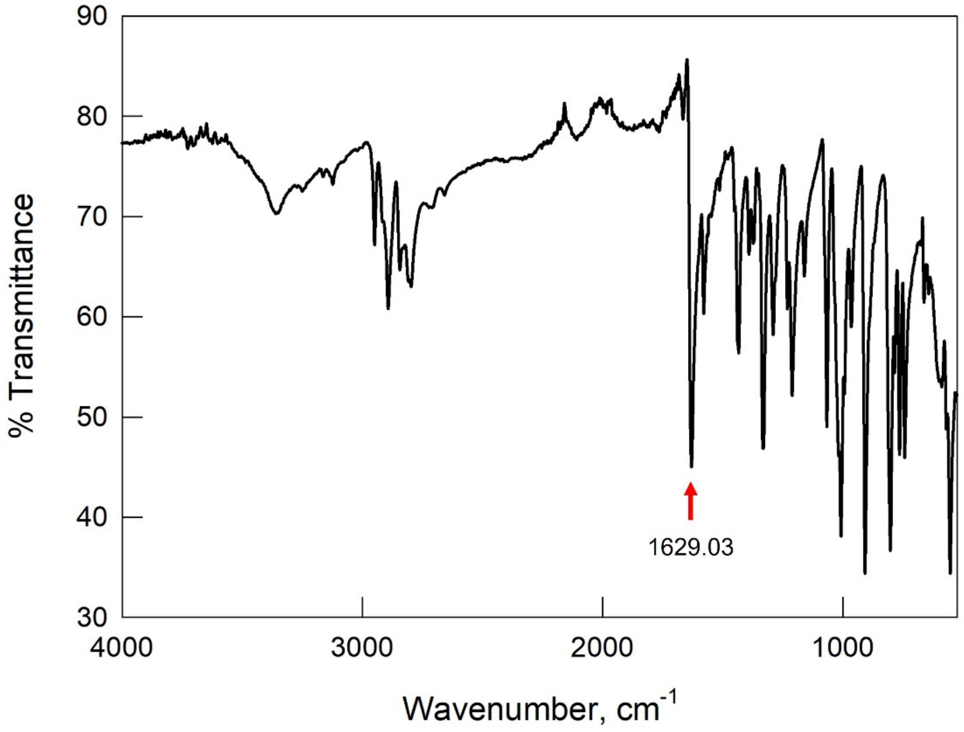

Figure S5. FTIR-ATR spectrum of Fura.

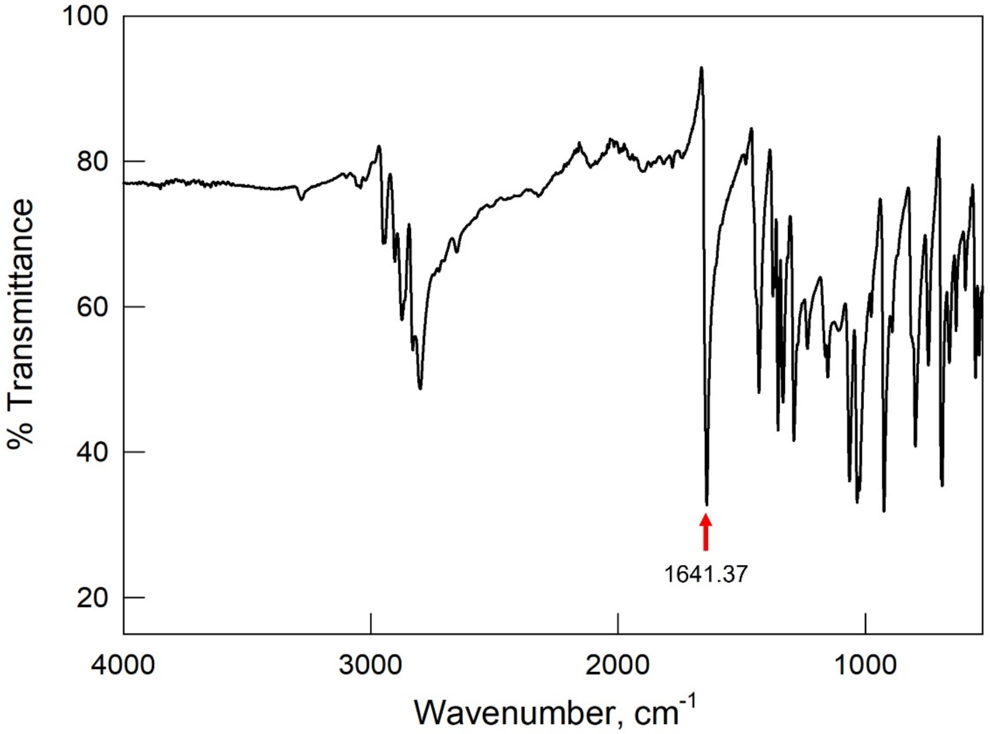

Figure S6 FTIR-ATR spectrum of m-xy.

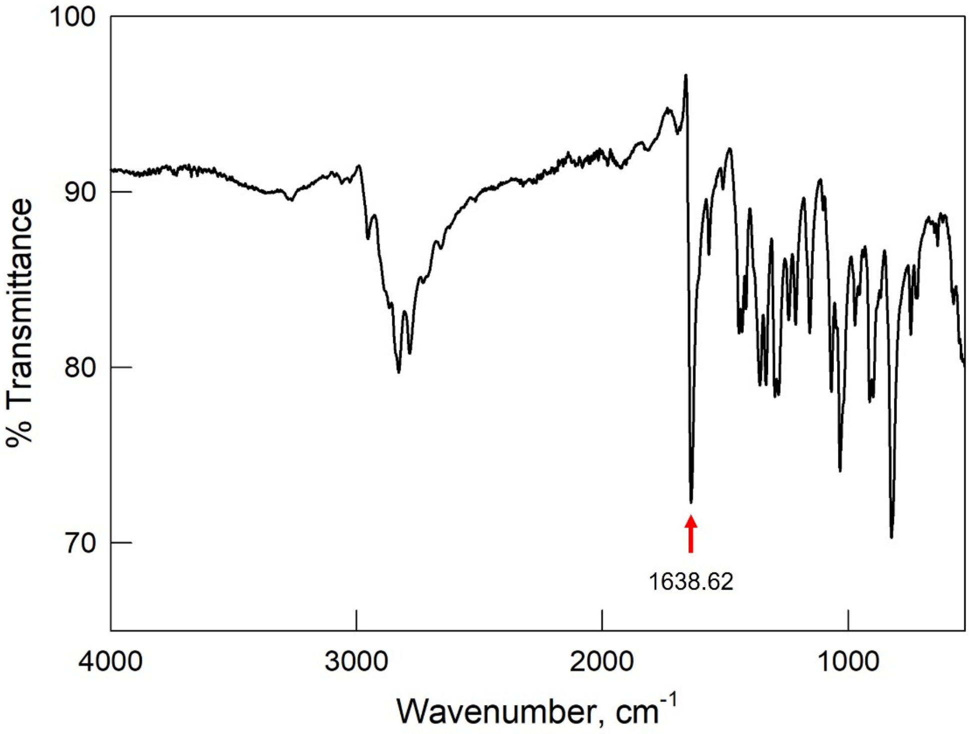

Figure S7 FTIR-ATR spectrum of p-xy

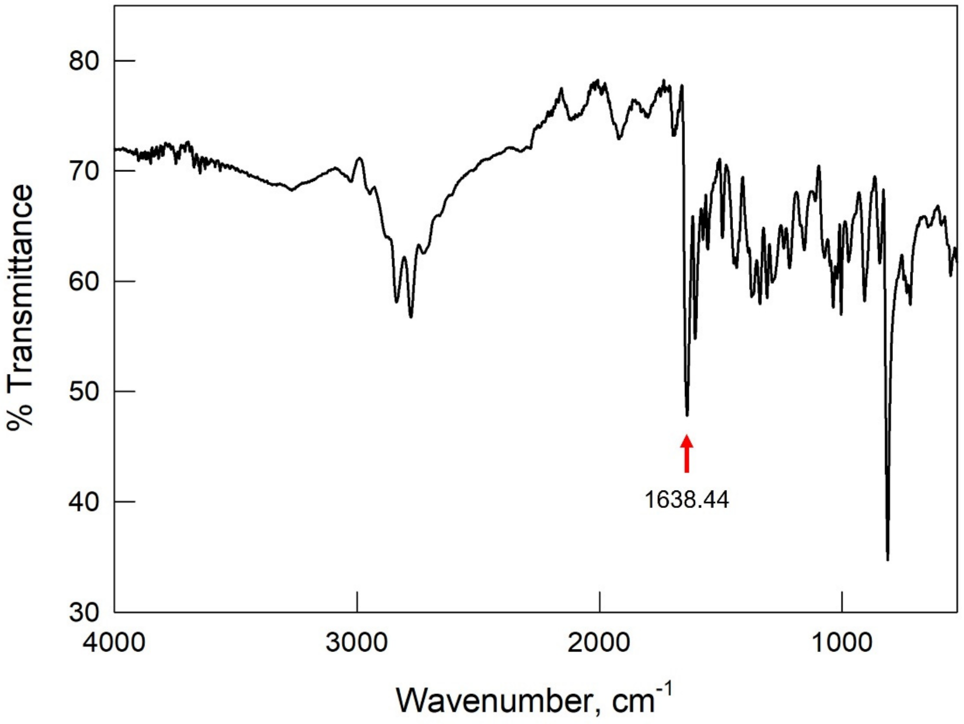

Figure S8 FTIR-ATR spectrum of the diphenyl cage.

2. Differential scanning calorimetric (DSC) measurements

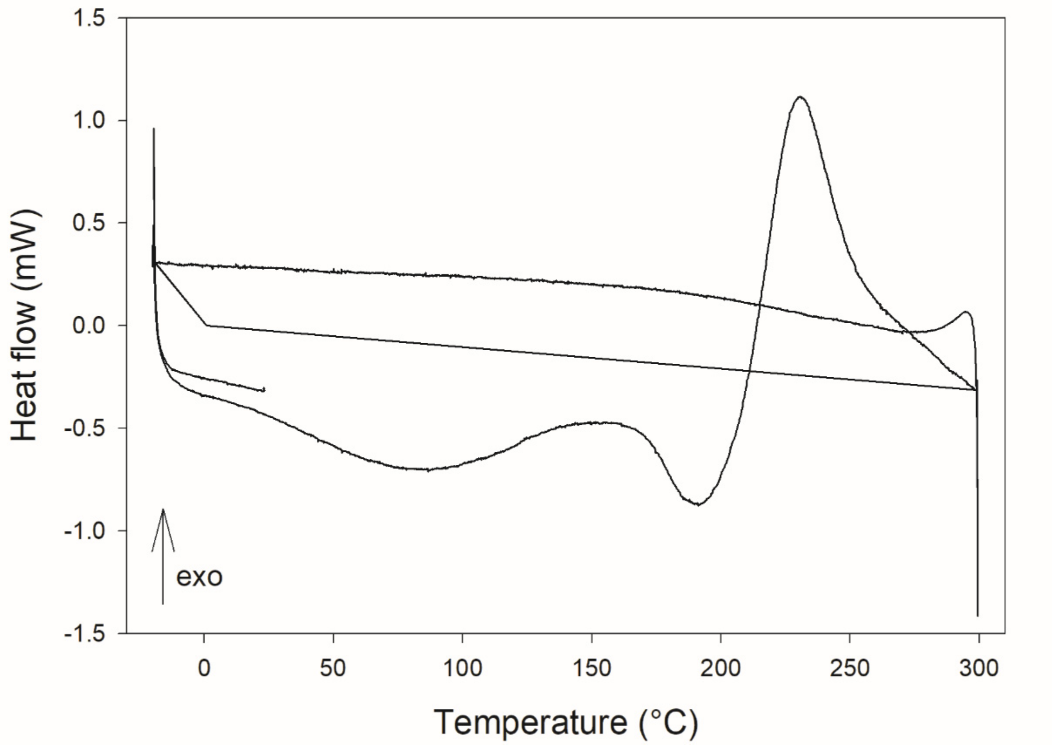

Figure S9 DSC curve of Fura.

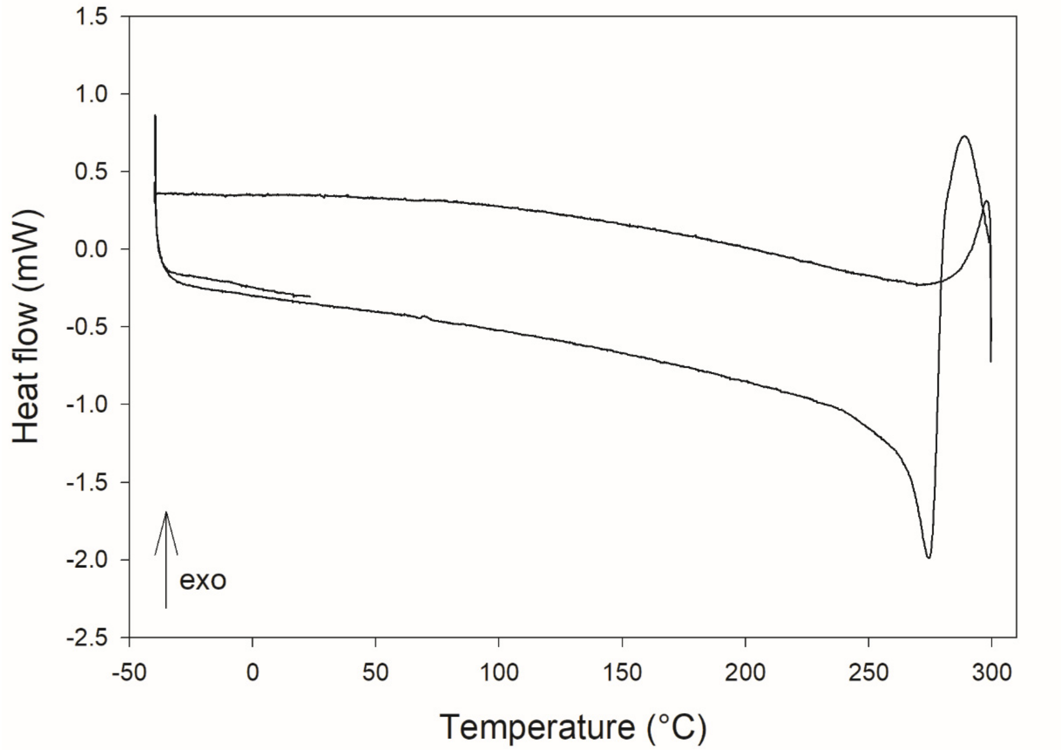

Figure S10 DSC curve of m-xy

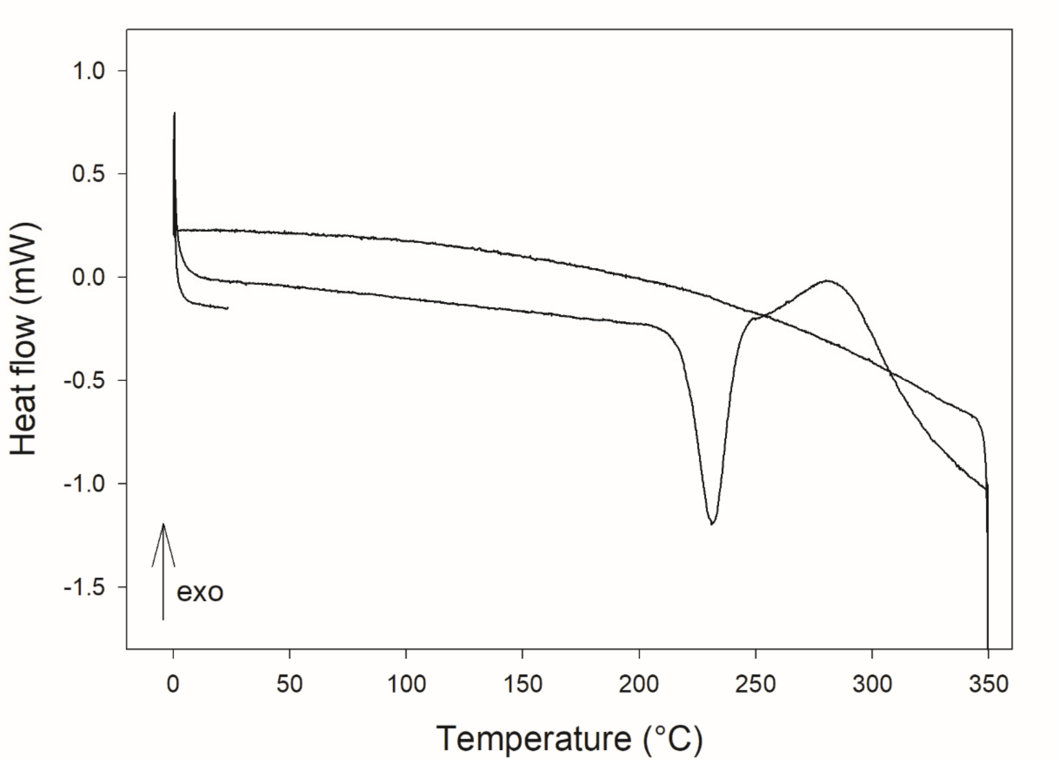

Figure S11 DSC curve of p-xy.

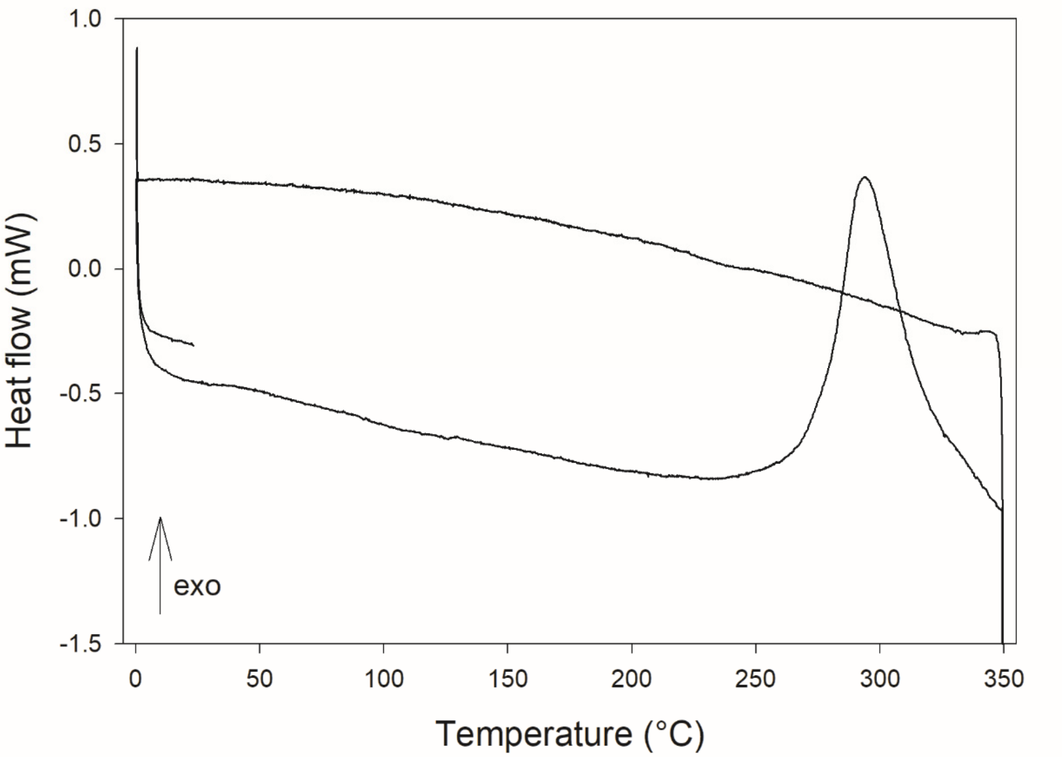

Figure S12 DSC curve of the diphenyl cage.
